# Superior physical and mental health of healthy volunteers before and five years after mobilized stem cell donation

**DOI:** 10.1186/s12967-022-03322-w

**Published:** 2022-03-14

**Authors:** J. Heyn, S. Bräuninger, M. Dimova-Dobreva, N. Mathieson, N. Koptelova, A. Kolpakova, C. Seidl, P. Reinhardt, C. Tsamadou, H. Schrezenmeier, R. Nakov, E. Seifried, H. Bonig

**Affiliations:** 1 Hexal AG (a Sandoz Company), Holzkirchen, Germany; 2 German Red Cross Blood Service Baden-Württemberg-Hessen, Institute Frankfurt, Frankfurt, Germany; 3 Institut für Klinische Transfusionsmedizin und Immungenetik Ulm (IKT), Ulm, Germany; 4 Institute for Transfusion Medicine and Immunohematology, Goethe University, Haus 76 Sandhofstr. 1, 60528 Frankfurt a.M., Germany; 5 Division of Hematology, Department of Medicine, University of Washington, Seattle, WA USA

**Keywords:** Healthy stem cell donors, Filgrastim, Physical and mental health, SF-12

## Abstract

**Background:**

Safety, tolerability and efficacy of granulocyte colony-stimulating factor (G-CSF) for mobilization of hematopoietic stem and progenitor cells (HSPCs) from healthy donors have been conclusively demonstrated. This explicitly includes, albeit for smaller cohorts and shorter observation periods, biosimilar G-CSFs. HSPC donation is non-remunerated, its sole reward being “warm glow”, hence harm to donors must be avoided with maximal certitude. To ascertain, therefore, long-term physical and mental health effects of HSPC donation, a cohort of G-CSF mobilized donors was followed longitudinally.

**Methods:**

We enrolled 245 healthy volunteers in this bi-centric long-term surveillance study. 244 healthy volunteers began mobilization with twice-daily Sandoz biosimilar filgrastim and 242 underwent apheresis after G-CSF mobilization. Physical and mental health were followed up over a period of 5-years using the validated SF-12 health questionnaire.

**Results:**

Baseline physical and mental health of HSPC donors was markedly better than in a healthy reference population matched for ethnicity, sex and age. Physical, but not mental health was sharply diminished at the time of apheresis, likely due to side effects of biosimilar G-CSF, however had returned to pre-apheresis values by the next follow-up appointment after 6 months. Physical and mental health slightly deteriorated over time with kinetics reflecting the known effects of aging. Hence, superior physical and mental health compared to the general healthy non-donor population was maintained over time.

**Conclusions:**

HSPC donors are of better overall physical and mental health than the average healthy non-donor. Superior well-being is maintained over time, supporting the favorable risk–benefit assessment of volunteer HSPC donation.

*Trial registration* National Clinical Trial NCT01766934

**Supplementary Information:**

The online version contains supplementary material available at 10.1186/s12967-022-03322-w.

## Introduction

A broad panel of immunodeficiencies, bone marrow failure syndromes and refractory hematological malignancies are treated with allogeneic HSPC transplantation [1]. For the majority thereof, G-CSF-mobilized peripheral blood HSPCs are the preferred graft source. Besides originator G-CSF, several biosimilars are available for HSPC mobilization [2, 3].

Biosimilars are biologics matching their reference biologic in terms of safety, efficacy and quality [4]. Similarity needs to be demonstrated through biochemical characterization, biologic activity in vitro, and clinical similarity in a sensitive indication [5, 6]. Once similarity is shown, the reference’s positive risk–benefit profile is assumed for the biosimilar [2, 7]. G-CSF biosimilars are thus approved for most indications for which the reference is licensed, of relevance to this work including HSPC mobilization in patients and donors [2, 8].

Efficacy and safety of G-CSF in healthy donors have been documented [9–11], including for biosimilar G-CSF [2]. Effects of HSPC donation on long-term physical and mental well-being, however, have not been explored. We therefore specifically analyzed long-term effects of G-CSF on mental and physical health of HSPC donors.

## Methods

### Study design

EP06-501 is a two-center non-interventional, long-term safety data collection of Zarzio/Filgrastim HEXAL in Stem Cell Donors (SMART; clinicaltrials.gov NCT01766934). The study was approved by the local ethics committees (approval #385/10). Inclusion criteria were those defining eligibility for G-CSF mobilized, unrelated donor HSPC donation according to World Marrow Donor Association; access to the study was restricted to donors from the registry “Deutsche Stammzellspenderdatei” (German Stem Cell Donor Registry, DSSD). Specification of donor evaluation and clearance were previously reported [12]. All participating donors fulfilled donor eligibility criteria and were cleared for donation. Donors provided separate written informed consent for mobilized donation and participation in this non-interventional long-term follow-up study.

HSPCs were mobilized with SANDOZ filgrastim biosimilar (Zarzio) at doses ≤ 10 µg/kg*day (dosed to the nearest full syringe) by self-injection in two divided subcutaneous doses. Donors who had consented to stem cell donation but not to participation in this non-interventional long-term follow-up study were mobilized according to the same protocol, donated stem cells using the same technology, and were offered participation in the identical (except for the SF12 questionnaire) regular donor follow-up provided by the DSSD; their outcome data are accordingly not reported here. This trial enrolled participants at German Red Cross Blood Service Baden-Württemberg-Hessen stem cell donor locales in Frankfurt (Center 1) and Ulm (Center 2).

Donors from Center 1 received the first dose in the morning of Day-4 and were collected after the ninth injection. At Center 2 donors started with the first dose in the evening of Day-4 and were consequently collected after eight doses. Two to four hours after the eighth (or ninth) dose, apheresis was initiated (morning of day zero) via peripheral venous access using conventional apheresis equipment (Spectra Optia MNC; Terumo BCT, Lakewood, CO) as reported previously [5]. Based on the concentration of circulating CD34+ cells, empirical algorithms were used to predict the necessary process volume to collect the requested dose of CD34+ cells with 99.5% probability. Termination of apheresis was done either when the required volume was reached or after a maximal duration of the apheresis of 300 min. Where the standard dose of 4 × 106 CD34 + cells/kg of the recipient had not been collected within the first apheresis, two additional doses of G-CSF were administered, and an additional apheresis was done (Day + 1).

No more than two aphereses and no more than 11 doses of G-CSF were routinely given (two donors received 12 doses due to donor error) according to standard operating procedures of the apheresis centers, backed by the Hemotherapy Guidelines of the German Medical Association, irrespective of the collected total stem cell dose [13].

Severity of bone pain (most frequent adverse effect of G-CSF) was assessed before each apheresis. A visual analog scale from 0 to 10 was used to quantify the average and maximal bone pain. A laboratory panel was analyzed at the time of donor assessment; before apheresis; at 1, 6, and 12 months after apheresis and will be analyzed annually thereafter up to year 10. Open questionnaires were used to capture adverse events (AEs). Severe AEs (SAE) were in accordance with the Good Clinical Practice definition. Physicians from the participating donor centers determined relatedness of an AE to G-CSF treatment.

Efficiency of mobilization (CD34+ cells/mL blood) as well as the CD34+ cell dose in the apheresis product was analyzed using flow cytometry (according to the European Directorate for the Quality of Medicines and Health Care) [14]. Standard automated laboratory equipment in accredited laboratories was used for the assessment of all other laboratory parameters.

The SF-12 questionnaire, which measures functional health and well-being from the donor's point of view, was applied at each visit to provide longitudinal data. SF-12 consists of twelve items from the SF-36 Health Survey [15] and covers the same eight domains of health outcomes, including physical functioning, role-physical, bodily pain, general health, vitality, social functioning, role-emotional, and mental health. The first four categories describe physical health, the four remaining categories overall mental health. Scores for both dimensions are transformed to range from 0 to 100, higher values reflecting more favorable conditions. SF-12 is validated for longitudinal re-test reliability [16]. Cohorts of relevant control populations are available for comparison. To account for cultural, medical and living conditions, two previously reported cohorts of self-reportedly healthy Germans were used as reference populations, throughout the manuscript they are referred to as “reference population” or “non-donors”. Since the reference population shows a relevant decline in physical health over age, as well as relevantly poorer mental well-being in females than in males, our donors were sorted according to the age brackets selected by the authors of the two reference populations as well as by sex, and well-being of donors and the general population (non-donors) was compared. Thereby, we sought to account for the apparently “physiological” decline especially of physical health that might confound potential changes over time in the donor population. During the five-year follow-up, some of the donors “aged out” of their initial age cohort and had to be re-assigned to the next older cohort. The reference cohorts being cross-sectional cohorts, i.e. not being followed over time, are the same for the analysis at the time of donation and 5 years thereafter.

Characteristics at screening were descriptively summarized using frequencies for categorical, mean/standard deviation/median/range for continuous variables. SF-12 scores for mental and physical health over time are also descriptively summarized. Statistical analyses were performed using SAS (Version 9.2, SAS Institute, Cary, NC). As applicable, t-test, Fisher’s exact test, Chi^2^-test, and two-sided one-sample binomial test were used. A p-value ≤ 0.05 was considered statistically significant.

## Results

During the study period, 242 healthy donors were included in the study. To mobilize HSPCs, all participants received filgrastim and no more than two aphereses were performed in each donor. Demographic characteristics are presented in Table 1.

**Table 1:** Demographic characteristics of completers and non-completers after 5 years

	Completers (n = 145)	Non-completers (n = 97)	Total (n = 242)	p-value
Gender, n (%)				
Male	109 (75.2)	71 (73.2)	180 (74.4)	0.765*
Female	36 (24.8)	26 (26.8)	62 (25.6)	
Age groups, n (%)^#^				
≤ 17	–	–	–	
18 to ≤ 19	–	1 (1.0)	1 (0.4)	
20 to ≤ 29	36 (24.8)	34 (35.1)	70 (28.9)	
30 to ≤ 39	51 (35.2)	29 (29.9)	80 (33.1)	
40 to ≤ 49	46 (31.7)	26 (26.8)	72 (29.8)	
50 to ≤ 59	12 (8.3)	6 (6.2)	18 (7.4)	
60 to ≤ 69	–	1 (1.0)	1 (0.4)	
> 70	–	–	–	
Age [years]^#^				
Mean ± SD	36.7 ± 9.6	34.7 ± 10.0	35.9 ± 9.8	0.128**
Median (IQR)	35 (30–45)	32 (27–43)	34 (28–43)	
BMI [kg/m^2^]^#^				
Mean ± SD	27.2 ± 4.5	26.6 ± 4.4	27.0 ± 4.4	0.247**
Median (IQR)	26.3 (24.2–29.4)	25.6 (23.5–28.7)	25.9 (24.0–29.4)	
Physical health score				
Mean ± SD	55.9 ± 5.1	56.3 ± 4.0	56.0 ± 4.7	0.529*
Median (IQR)	57.1 (54.9–58.4)	57.1 (55.0–58.4)	57.1 (55.0–58.4)	
Mental health score				
Mean ± SD	55.7 ± 5.1	54.6 ± 5.6	55.3 ± 5.3	0.146*
Median	56.9 (54.5–58.8)	56.0 (51.8–57.5)	56.8 (53.2–57.9)	

The given mental and physical scores were collected at screening by using the SF-12 questionnaire. No statistical differences were found between 5-year completer and non-completer in terms of gender, age, BMI, physical and mental health. SD: Standard deviation, IQR: interquartile range, BMI: body mass index

^*^Fisher's exact test; ** t-test; # at the time of mobilization/apheresis; Physical/Mental health scores were only available for 142 completers and 96 non-completers

Since there is still an ongoing debate if G-CSF may influence on the occurrence of malignancies, we first analyzed the incidence of malignancies in our study population and compared it with the general German population. Although two cases of malignant melanoma and one case each of Hodgkin’s lymphoma and thyroid cancer statistically exceed the expected incidence for a cohort the size of ours (Table 2), post hoc power analysis for the comparison of incidence rates showed that the power is not sufficient to show a significant difference between both groups for this and similar health outcomes (Additional file 1: Table S1).

**Table 2:** Incidence of malignancies during follow-up

	Observed number of cases in the study population	Annualized incidence rate in Germany (per 100.000)	Expected number of cases in the study population	p-value
Malignant skin melanoma	2	24.22	0.193	**< 0.0001**
Hodgkin's lymphoma	1	2.89	0.023	**< 0.0001**
Non-Hodgkin's lymphoma	0	20.78	0.166	0.6835
Multiple myeloma	0	9.42	0.075	0.7837
Myelodysplastic, myeloproliferative, and other hematopoietic neoplasms	0	4.40	0.035	0.8512
Thyroid cancer	1	3.02	0.023	**< 0.0001**

Statistically significant differences are shown in boldscript

Comparison of incidence rates of malignancies during the study with those of the German population in 2019 (using a Chi^2^-test). Based on the incidence rate of the different diseases for the German population according to the German cancer registry the expected number for our cohort is calculated over the cumulative observation time corrected for the time of discontinuation, if applicable, and assuming largely age-independent, linear distribution of cancer occurrence [27]

**Fig. 1:**
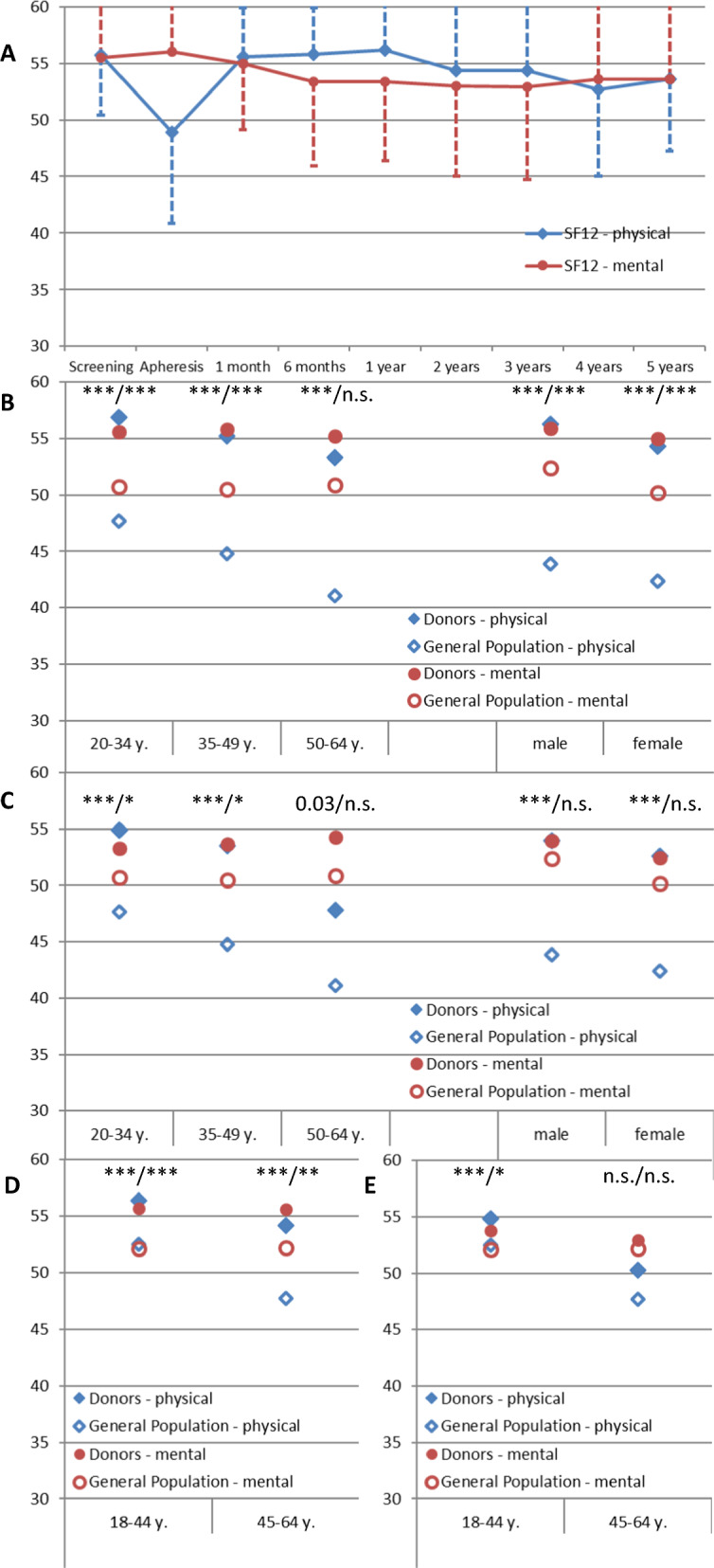
Well-being over time as assessed with the SF-12 self-guided questionnaire. **A** Physical and mental well-being (Y-axis) over the phases of screening, apheresis and follow-up (X-axis) are displayed (mean, SD; for legibility, SD is drawn in only one direction). A dramatic transient dip in physical well-being is observed after mobilization with G-CSF, assessed just prior to the first apheresis, as well as a slow but steady decline of both physical and mental health scores is seen over time. **B**, **C** Age- (left) and sex-(right) adjusted physical and mental well-being of donors compared to a healthy cohort of German non-donors (published by Kuehne et al. [22]) at the time of donor screening (**B**) and at the five-year follow-up time point (**C**). **D**, **E** Age-adjusted physical and mental well-being of donors compared to a healthy cohort of German non-donors (published by Gandek et al.[23]) at the time of donor screening (**D**) and at the five-year follow-up time point (**E**). For both comparisons, donors were assigned to the respective age group based on their age at this specific time-point (i.e. at the time of donation, of 5-year follow-up). Of note, the Gandek cohort contains equal numbers of women and men, whereas our cohort is significantly skewed towards men who generally score higher on the SF-12 for both physical and mental health status. The data in panels **B**–**E** show a markedly better than expected health status of HSPC donors compared to healthy non-donors of all age groups and for both sexes; the advantage is largely maintained over time. Throughout, physical and mental well-being are depicted as blue diamonds and red circles, respectively. Values for donors from our cohort are displayed as full symbols, those for the published reference groups as empty symbols. Asterisks indicate statistical significance at the ≤ 0.0001 (***), ≤ 0.001 (**), ≤ 0.01 (*) level, values between 0.01 and 0.05 are shown, n.s. (not significant) indicates a p > 0.05

Next, we focused on the long-term effect of HSPC donation on physical and mental health. Our study is unique among all previously performed donor outcome studies in that physical and mental health is formally quantitatively assessed using validated sociological tools. The SF-12 questionnaire was applied at each visit to measure these factors from the donor's perspective. Similar baseline scores for physical and mental health were observed, followed by a transient dip only in physical well-being after mobilization with G-CSF, assessed just prior to the first apheresis, then a slow but steady decline of both physical and mental health scores over the observation time (Fig. 1A). Higher baseline and 5-year scores were associated with younger age and male sex. Before mobilization and donation, donors, especially younger donors, had significantly higher scores for both mental and physical health compared to two age-adjusted cohorts of healthy Germans (Fig. 1B, D). Higher scores are maintained throughout the observation period irrespective of the age at donation, for a minimum of 5 years (Fig. 1C, E).

Since healthy donors show a transient dip in physical health, we analyzed if the most common AEs at apheresis might be associated with lower physical health scores. Indeed, significantly more strongly reduced physical health scores are observed in donors experiencing bone pain (physical health score in patients with bone pain: 48.2 ± 8.5) when compared to those without bone pain (physical health score in patients without bone pain: 53.9 ± 4.8, p < 0.05). None of the other more common AEs had a significant association with the physical health score (Additional file 1: Table S2). To gauge whether adverse events during the post-mobilization period might affect willingness to provide long-term follow-up and hence, skew our analyses, we analyzed type and frequency of adverse events after the mobilization period in participants who complete the five-year period and those who did not. There was a trend in favor of patients with serious adverse events, i.e. the overall favorable outcome is observed in spite of a selection bias for donors with health events (Table 3). Finally, we compared the SF-12 scores one month after apheresis in donors subjected to one versus two aphereses to ask whether providing two consecutive apheresis was associated with measurably different, presumably lower, health scores. We did not find significant differences in physical (one apheresis: 55.2 ± 4.9, two aphereses: 55.7 ± 1.8; p = 0.682) and mental health score (one apheresis: 54.9 ± 6.6, two aphereses: 54.0 ± 5.5; p = 0.628) between both groups (Additional file 1: Table S3).

**Table 3:** Association of adverse events and long-term participation in the study

	Completer n = 145	Non-completer n = 97	All donors n = 242	p-value
Any AE	88 (60.7%)	37 (38.1%)	125 (51.7%)	
	[52.2–68.7]	[28.5–48.6]	[45.2–58.1]	
Any serious AE	31 (21.4%)	7 (7.2%)	38 (15.7%)	0.089
	[15.0–29.0]	[3.0–14.3]	[11.4–20.9]	
AE related to G-CSF treatment	12 (8.3%)	2 (2.1%)	14 (5.8%)	0.228
	[4.3–14.0]	[0.3–7.3]	[3.2–9.5]	
Serious AE related to G-CSF treatment	3 (2.1%)	0 (0.0%)	3 (1.2%)	0.554
	[0.4–5.9]	[0.0–3.7]	[0.3–3.6]	
AE of special interest	2 (1.4%)	0 (0.0%)	2 (0.8%)	1.000
	[0.2–4.9]	[0.0–3.7]	[0.1–3.0]	
AE related to apheresis procedure	0 (0.0%)	1 (1.0%)	1 (0.4%)	0.296
	[0.0–2.5]	[0.0–5.6]	[0.0–2.3]	

We hypothesized that adverse events in the post-mobilization period might negatively affect willingness to provide long-term follow-up. In order to address a possible connection we analyzed the occurrence of adverse events after the mobilization period in participants who complete 5 years and those who did not (using Fisher’s exact test). Donors experiencing any serious AE were, if anything, over-represented in the completer cohort (not statistically significant). Frequency is given in round brackets, interquartile range in square brackets. AE: adverse events, SAE: serious adverse events

## Discussion

We had previously reported that mobilization with biosimilar G-CSF was efficient as well as acutely safe with no unexpected or limiting adverse events [5]. Analyses of 5-year outcomes do not change this impression. Attributable (severe) adverse events outside the immediate mobilization/post-mobilization period were not detected. Potential long-term effects of G-CSF administration, specifically a contribution to cancer, has fueled ongoing debates [17–19]. Therefore, we analyzed the limited number of malignancies and could demonstrate that attributability is not suspected, the excess in incidence likely random. Further malignancies were not observed, specifically not myeloid leukemias/myelodysplasia a causal association of which with G-CSF could possibly be construed. Clearly, due to the small cohort size the power to detect subtle differences is limited. Our findings corroborate much larger earlier studies supporting long-term safety of healthy-donor G-CSF stimulation [9, 20].

The distinguishing, indeed unique feature of our long-term donor follow-up is the formal longitudinal assessment of physical and mental health using validated sociological tools. The sensitivity of the SF-12 questionnaire to catch changes in physical or mental health in longitudinal analyses is well documented [10, 21]. Our observations confirm this notion for the physical health score: The association of lower scores with the mobilization period is apparent. This transient dip in perceived physical health is likely causally related to prevalent, intervention-inherent adverse events, especially pain that the SF-12 explicitly queries and thus sensitively detects. Before mobilization and donation, donors, especially younger donors, had significantly higher scores for both mental and physical health referenced against two independent age-adjusted cohorts of (self-reportedly) healthy German non-donors [22, 23]. As to the reasons for this observation, we can only speculate. We propose that a significant contributor likely is selection bias, both self-selection, whereby individuals with (perceived) superior physical and mental health may be more likely to register as donors, and selection during donor eligibility testing [12]. Moreover, absent financial incentives for registration as stem cell donor and stem cell donation, the only driving force for stem cell donors is altruism. It is reasonable to assume that such a positive attitude pervades the personality, is not restricted to the act of donation and is a sustained positive risk factor especially for superior mental well-being. We further propose that overall, donors may be more body conscious, take better care of their health including leading, on average, relatively healthier life-styles. A substantial body of work has studied motivations for and well-being of blood donors, a markedly less invasive form of donation. In agreement with our hypotheses for stem cell donors, selection bias (only healthy persons being allowed to become donors [24]) and altruism, as well as the belief that blood donation provides health benefits [25] have been mentioned. Importantly, higher scores are maintained throughout the observation period, for a minimum of 5 years, i.e. G-CSF mobilization and HSPC donation are not associated with accelerated deterioration of physical or mental health. For one, the earlier discussed selection biases likely continue to be relevant, as well as the positive personality traits (the “happy donor” effect) inducing an individual to “share the gift of life” with a stranger are likely sustained. Secondly, we cannot exclude the possibility that the act of donation itself imprints or reinforces positive, health-adaptive attitudes. Indeed, evidence has been provided that positive events such as a positive donor experience have long-term beneficial effects [26]. Finally, the donor experience may have reminded the donor of the value of good health and have induced or re-enforced healthy habits or life-styles, especially since the donors are informed during donor evaluation about the possibility of additional donations for the same patient in the future, so that a sense of responsibility towards “their” patient may further incentivize the donor to maintain a healthy life-style. To ascertain whether the positive health outcomes were not a subject of post-donation selection bias, we additionally compared donors completing or not follow-up with respect to well-being, probability to suffer adverse events or undergoing two-day apheresis. Neither affected the probability of providing long-term follow-up nor baseline SF-12 scores, thus the followed-up cohort is representative of the complete study population and possibly, G-CSF-mobilized donors per se.

The choice and limitations of the reference cohorts also merit discussion. Unlike the heavily selected donor cohort, these represent a self-reportedly healthy, but not nearly as stringently characterized population. In this sense, some of the superior well-being of the donor cohort, especially in the physical domain, should probably not come as a surprise. Another potentially meaningful difference between the donor and reference cohorts is the longitudinal nature of the former, cross-sectional nature of the latter. However, the demonstrable re-test reliability of the SF12 implies that this effect should be relatively subtle. Significant cultural effects of SF12 scores have been acknowledged; this ethno-cultural bias is accounted for by the selected reference populations. The ideal comparator for our donor population, an age- and sex-matched cohort of similarly selected individuals not proceeding to stem cell donation but similarly followed over time, was not available.

Our data corroborate long-term safety of (biosimilar) G-CSF for healthy-donor HSPC mobilization. The unique, previously not performed longitudinal analysis of physical and mental health with validated health assessment scores demonstrates strongly superior health of HSPC donors before and at all times after mobilization, formally underscoring absence of long-term adverse health outcomes after HSPC donation.

## Supplementary Information

**Additional file 1**: A supplement with additional detail about the study design (Supplemental methods) and tables comparing obeserved and expected case number for malignant tumors (**Table S1**), power analysis for detection of differences in cancer incidence (**Table S2**), analysis of association between likelihood to suffer AEs and QOL (**Table S3**), analysis of association of probability of suffering AEs and continued study participation (**Table S4**), analysis of association of apheresis number and QOL (**Table S5**). The supplement contains supplemental references.
