# Supplementary material for: Superior physical and mental health of healthy volunteers before and five years after mobilized stem cell donation
Source: J Transl Med. 2022 Mar 14;20:121. doi: 10.1186/s12967-022-03322-w (PMC8919626; doi:10.1186/s12967-022-03322-w)
Supplement: Supplementary file 1 — Additional file 1: A supplement with additional detail about the study design (Supplemental methods) and tables comparing obeserved and expected case number for malignant tumors (Table S1), power analysis for detection of differences in cancer incidence (Table S2), analysis of association between likelihood to suffer AEs and QOL (Table S3), analysis of association of probability of suffering AEs and continued study participation (Table S4), analysis of association of apheresis number and QOL (Table S5). The supplement contains supplemental references. [file 12967_2022_3322_MOESM1_ESM.docx]

**Supplemental Methods**

**Study design**

Nonconsenting donors were mobilized by the same protocol, donated stem cells using the same technology, and were offered participation in the identical regular donor follow-up provided by the German Stem Cell Donor Registry. This trial enrolled participants at German Red Cross Blood Service Baden-Württemberg-Hessen stem cell donor locales in Frankfurt (Center 1) and Ulm (Center 2).

Donors from Center 1 received the first dose in the morning of Day -4 and were collected after the ninth injection. At Center 2 donors started with the first dose in the evening of Day -4 and were consequently collected after eight doses. Two to four hours after the eighth (or ninth) dose, apheresis was initiated (morning of day zero) via peripheral venous access using conventional apheresis equipment (Spectra Optia MNC; Terumo BCT, Lakewood, CO) as reported previously.^1^ Based on the concentration of circulating CD34^+^ cells, empirical algorithms were used to predict the necessary process volume to collect the requested dose of CD34^+^ cells with 99.5% probability. Termination of apheresis was done either when the required volume was reached or after a maximal duration of the apheresis of 300 minutes. Where the standard dose of 4x10^6^ CD34^+^ cells/kg of the recipient had not been collected within the first apheresis, two additional doses of G-CSF were administered, and an additional apheresis was done (Day +1).

No more than two apheresis and no more than 11 doses of G-CSF were routinely given (two donors received 12 doses due to donor error) according to standard operating procedures of the apheresis centers, backed by the Hemotherapy Guidelines of the German Medical Association, irrespective of the collected total stem cell dose.^2^

Severity of bone pain (most frequent adverse effect of G-CSF) was assessed before each apheresis. A visual analog scale from 0 to 10 was used to quantify the average and maximal bone pain. A laboratory panel was analyzed at the time of donor assessment; before apheresis; at 1, 6, and 12 months after apheresis and will be analyzed annually thereafter up to year 10. Open questionnaires were used to capture adverse events (AEs). Severe AEs (SAE) were in accordance with the Good Clinical Practice definition. Physicians from the participating donor centers determined relatedness of an AE to G-CSF treatment.

Efficiency of mobilization (CD34^+^ cells/mL blood) as well as the CD34^+^ cell dose in the apheresis product was analyzed using flow cytometry (according to the European Directorate for the Quality of Medicines and Health Care).^3^ Standard automated laboratory equipment in accredited laboratories was used for the assessment of all other laboratory parameters.

**Table S1: Incidence of malignancies during follow-up**

Comparison of incidence rates of malignancies during the study with those of the German population in 2019 (using a Chi^2^-test). Based on the incidence rate of the different diseases for the German population according to the German cancer registry the expected number for our cohort is calculated over the cumulative observation time corrected for the time of discontinuation, if applicable, and assuming largely age-independent, linear distribution of cancer occurrence.^4^

|  | **Observed number of cases in the study population** | **Annualized incidence rate in**  **Germany (per 100.000)** | | **Expected number**  **of cases in the study population** | | **p-value** | |  |
| --- | --- | --- | --- | --- | --- | --- | --- | --- |
| Malignant skin melanoma | 2 | 24.22 | 0.193 | |  |  | **<0.0001** | |
| Hodgkin's lymphoma | 1 | 2.89 | 0.023 | |  |  | **<0.0001** | |
| Non-Hodgkin's lymphoma | 0 | 20.78 | 0.166 | |  |  | 0.6835 | |
| Multiple myeloma | 0 | 9.42 | 0.075 | |  |  | 0.7837 | |
| Myelodysplastic, myeloproliferative, and other hematopoietic neoplasms | 0 | 4.40 | 0.035 | |  |  | 0.8512 | |
| Thyroid cancer | 1 | 3.02 | 0.023 | |  |  | **<0.0001** | |

**Table S2: Power analysis for ability to detect differences in cancer incidence between donors and the general population**

Post hoc power analysis for the comparison of incidence rates shows that the power is not sufficient to show a significant difference between both groups (alpha error 0 0.05; power = 0.95).

| **Disease** | **Incidence rate in Germany** | |  | **Post hoc power** |
| --- | --- | --- | --- | --- |
| Malignant skin melanoma | 24.22 |  |  | 0.595 |
| Hodgkin's lymphoma | 2.89 |  |  | 0.632 |
| Non-Hodgkin's lymphoma | 20.78 |  |  | 0.044 |
| Multiple myeloma | 9.42 |  |  | 0.010 |
| Myelodysplastic, myeloproliferative, and other hematopoietic neoplasms | 4.40 |  |  | 0.125 |
| Thyroid cancer | 3.02 |  |  | 0.632 |

**Table S3: Association of mobilization-related adverse events and SF-12 physical health score at the time of apheresis**

SF-12 physical health score at apheresis in donors with certain AEs compared to donors not experiencing these AEs. A significant reduction in physical health score is observed in donors experiencing bone pain, which is an almost universal, albeit highly transient adverse effect of filgrastim in healthy donors.
**AE:** adverse event; **SD:** Standard deviation.

| **Donors with AE** | | | | **Donors without AE** | | | |
| --- | --- | --- | --- | --- | --- | --- | --- |
| **Specific AE** | **n** | **Mean** | **SD** | **n** | **Mean** | **SD** | **p-value** |
| Back pain | 35 | 47.6 | 9.44 | 203 | 48.5 | 8.30 | 0.5425 |
| Hypokalemia | 52 | 47.4 | 8.65 | 186 | 48.7 | 8.41 | 0.3241 |
| Bone pain | 229 | 48.2 | 8.51 | 9 | 53.9 | 4.75 | **0.0476** |
| Headache | 71 | 46.9 | 8.60 | 167 | 49.0 | 8.34 | 0.0759 |
| Pain in extremity | 19 | 49.3 | 8.30 | 219 | 48.3 | 8.49 | 0.6211 |
|  | | | | | | | |

**Table S4: Association of adverse events and long-term participation in the study**

We hypothesized that adverse events in the post-mobilization period might negatively affect willingness to provide long-term follow-up. In order to address a possible connection we analyzed the occurrence of adverse events after the mobilization period in participants who complete 5 years and those who did not (using Fisher’s exact test). Donors experiencing any serious AE were, if anything, over-represented in the completer cohort (not statistically significant). Frequency is given in round brackets, interquartile range in square brackets. **AE:** adverse events, **SAE:** serious adverse events.

|  | **Completer n=145** | | **Non-completer n=97** | **All donors n=242** | **p-value** |
| --- | --- | --- | --- | --- | --- |
| Any AE | 88 (60.7%) | 37 (38.1%) | | 125 (51.7%) |  |
|  | [52.2-68.7] | [28.5-48.6] | | [45.2-58.1] |  |
| Any serious AE | 31 (21.4%) | | 7 (7.2%) | 38 (15.7%) | 0.089 |
|  | [15.0-29.0] | | [3.0-14.3] | [11.4-20.9] |  |
| AE related to G-CSF treatment | 12 (8.3%) | | 2 (2.1%) | 14 (5.8%) | 0.228 |
|  | [4.3-14.0] | | [0.3-7.3] | [3.2-9.5] |  |
| Serious AE related to G-CSF treatment | 3 (2.1%) | | 0 (0.0%) | 3 (1.2%) | 0.554 |
|  | [0.4-5.9] | | [0.0-3.7] | [0.3-3.6] |  |
| AE of special interest | 2 (1.4%) | | 0 (0.0%) | 2 (0.8%) | 1.000 |
|  | [0.2-4.9] | | [0.0-3.7] | [0.1-3.0] |  |
| AE related to apheresis procedure | 0 (0.0%) | | 1 (1.0%) | 1 (0.4%) | 0.296 |
|  | [0.0-2.5] | | [0.0-5.6] | [0.0-2.3] |  |

**Table S5: Association of SF-12 score with number of aphereses**

Comparing SF-12 scores one month after apheresis in donors subjected to one versus two aphereses using t-test did not show any significant differences in physical and mental health score between both groups. SF-12 scores were available for 177 participants who had a single apheresis and 15 participants who had two aphereses. **SD:** standard deviation, **IQR:** interquartile range.

|  | **One apheresis (n=220)** | **Two aphereses (n=22)** | **Total (n=242)** | **p-value** |
| --- | --- | --- | --- | --- |
|  | | | | |
| **Physical health score** |  |  |  |  |
| Mean ± SD | 55.2 ± 4.9 | 55.7 ± 1.8 | 55.3 ± 4.7 | 0.682 |
| Median (IQR) | 57.1 (54.1/58.1) | 55.9 (54.3/57.1) | 57.1 (54.3/57.8) |  |
|  | | | | |
| **Mental health score** |  |  |  |  |
| Mean ± SD | 54.9 ± 6.6 | 54.0 ± 5.5 | 54.8 ± 6.5 | 0.628 |
| Median (IQR) | 57.4 (53.0/58.2) | 57.4 (52.4/57.5) | 57.4 (52.9/58.1) |  |

**Literature**

1. Becker P, Schwebig A, Brauninger S, et al. Healthy donor hematopoietic stem cell mobilization with biosimilar granulocyte-colony-stimulating factor: safety, efficacy, and graft performance. *Transfusion*. 2016;56(12):3055-3064.

2. National Medical Council. [Guidelines for collection of blood and blood components and for the application of blood products (hemotherapy)]. Köln (Germany): Deutscher Ärzteverlag; 2008.

3. Dauber K, Becker D, Odendahl M, Seifried E, Bonig H, Tonn T. Enumeration of viable CD34(+) cells by flow cytometry in blood, bone marrow and cord blood: results of a study of the novel BD stem cell enumeration kit. *Cytotherapy*. 2011;13(4):449-458.

4. <https://www.krebsdaten.de/Krebs/EN/Home/homepage_node.html>; Accessed 19 March 2021.
